# Insights into Bioactive Molecules in *Rhododendron tomentosum*: From Metabolomics to Biological Applications

**DOI:** 10.3390/biom16010110

**Published:** 2026-01-08

**Authors:** Giovanna Schiavone, Paola Imbimbo, Sabrina De Pascale, Rosalia Ferracane, Simonetta Caira, Andrea Scaloni, Antonio Dario Troise, Daria Maria Monti, Vincenzo Rocco, Daniela D’Esposito, Maurilia Maria Monti

**Affiliations:** 1 Department of Chemical Sciences, University of Naples Federico II, Complesso Universitario Monte Sant’Angelo, Via Cinthia 4, 80126 Naples, Italy; giovanna.schiavone@unina.it (G.S.); paola.imbimbo@unina.it (P.I.); 2 Institute for Sustainable Plant Protection, National Research Council, P.le E. Fermi 1, 80055 Portici, Italy; daniela.desposito@cnr.it (D.D.); mauriliamaria.monti@cnr.it (M.M.M.); 3 Institute for the Animal Production System in the Mediterranean Environment, National Research Council, P.le E. Fermi 1, 80055 Portici, Italy; sabrina.depascale@cnr.it (S.D.P.); rosaliaferracane@cnr.it (R.F.); simonetta.caira@cnr.it (S.C.); andrea.scaloni@cnr.it (A.S.); 4 CeMON S.r.l. Centro di Medicina Omeopatica Napoletano, Viale Gramsci 18, 80122 Naples, Italy; v.rocco@cemon.eu

**Keywords:** *Rhododendron tomentosum*, biological activities, metabolomics, bioactive compounds, natural products

## Abstract

*Rhododendron tomentosum* is an aromatic plant belonging to the Ericaceae family, widely used for different applications, but still lacking in its molecular signature. This work provides a complete chemical and biological characterization of the hydroalcoholic extract of *R. tomentosum* tips of twigs. Combining untargeted metabolomic analysis with bioassays, a correlation between chemical composition and biological activity was defined. To this regard, liquid chromatography high-resolution tandem mass spectrometry (LC-MS/MS) revealed a heterogeneous chemical composition, including flavonoids, such as quercetin, catechin, and their derivatives, as well as a first tentative identification of novel aesculin derivatives. Cell-based model experiments on stressed immortalized human keratinocytes demonstrated the antioxidant activity of the extract. Moreover, it exhibited significant antifungal and antibacterial effects against *Trichoderma atroviride* AGR2, *Botrytis cinerea*, and *Clavibacter michiganensis*, while promoting the growth of the beneficial bacterium *Bacillus amyloliquefaciens*. These findings highlight the rich diversity of bioactive molecules present in *R. tomentosum* hydroalcoholic extract, bridging its chemical composition to its functional properties. Overall, these results suggest its promising potential for applications in improving plant health, as well as in pharmaceutical, cosmetic, and agricultural industries.

## 1. Introduction

Plants are the primary source of bioactive metabolites with potential industrial application as sustainable alternatives to chemically synthetized products, as bioprotectants and biostimulants in agricultural systems, as well as a source of active molecules for pharmaceutical and cosmetic applications [1,2,3,4]. Phytometabolites have largely been investigated for their role in the plant defense system against herbivores, insects, and environmental stresses in agricultural systems [1,5]. Among them, essential oils have been largely investigated for their insecticidal, virucidal, fungicidal, bactericidal, and antiparasitic properties [6,7]. Despite the possible economic relevance, the large use of plant extracts to replace synthetic products is still poorly exploited.

*Rhododendron tomentosum*, formerly *Ledum palustre*, is an aromatic plant belonging to the Ericaceae family. It is widely spread in boreal forests, mires, and damp heaths of the Northern Hemisphere, such as Scandinavia, Ireland, and Canada. *R. tomentosum* is also known as wild rosemary, marsh tea or marsh rosemary, and Labrador tea [8]. To date, *R. tomentosum* extracts are normally obtained from the aerial parts of the plant and used in different forms, such as decoctions, purified essential oils, aqueous or oil infusion preparations, or hydroalcoholic extracts, with applications in the medical and insecticidal fields [9]. The anti-inflammatory and antiseptic properties of these preparations have been attributed to polyphenols and sesquiterpenes [10,11], whereas the insecticidal activity has been related to the composition of the essential oil [12,13], which has recently been proven to be toxic for humans depending on the dose [9].

To date, the literature on *R. tomentosum* chemical characterization relies on specific classes of compounds, such as coumarins and flavonoids [14,15,16,17], but a comprehensive metabolomic identification, along with a broad evaluation of its biological activities, is still lacking. Filling this gap could be particularly relevant, as phytochemicals hold significant potential for different industrial applications, including cosmetic formulations, natural antimicrobials, and environmentally friendly plant protection products. By integrating multiple analytical and biological approaches, the present study established the interconnection between chemical composition and functional activity of *R. tomentosum* extract.

## 2. Materials and Methods

### 2.1. Chemicals

All reagents, liquid chromatography mass spectrometry solvents, and the library of phytochemical standards were purchased from Merck-Sigma Aldrich (St. Louis, MO, USA), unless differently specified. The *R. tomentosum* hydroalcoholic extract was purchased by HERBAMED AG (Bühler, Switzerland). According to the manufacturer, plants were cultivated through wild harvesting in Ukraine, and tips of twigs were manually collected in June 2020. The extraction process was standardized according to German Homeopathic Pharmacopoeia (GHP). Briefly, the extract was prepared by percolating for 12 days dried tips of twigs with 70% ethanol and 30% H_2_O (*v*/*v*) in a ratio between biomass and solvent of 1:10 (*w*/*w*). Three hydroalcoholic extract batches were used.

### 2.2. Liquid Chromatography High-Resolution Tandem Mass Spectrometry (LC-MS/MS) Analysis

Aliquots of 1 mL of *R. tomentosum* extract were evaporated using N_2_, then dissolved in 1 mL of a methanol/water (70:30, *v*/*v*). The suspension was vortexed for 10 min and then centrifuged (18,000× *g*, 10 min, 4 °C). The supernatants were diluted ten times in a methanol/water mixture (70:30, *v*/*v*) and analyzed with a Vanquish Core liquid chromatographic system paired to an Exploris 120 quadrupole Orbitrap high-resolution mass spectrometer (both from Thermo Fisher Scientific, Bremen, Germany). Compounds were separated at 35 °C using a reversed-phase mode biphenyl Core–Shell column (Kinetex biphenyl, 100 × 2.1, 2.6 µm, Phenomenex, Torrance, CA, USA), eluted with 0.1% (*v*/*v*) formic acid in water (solvent A) and acetonitrile (solvent B), with the following gradient of solvent B: min 0–0.5, 5%; min 1.5, 15%; min 10, 40%; min 15, 70%; and min 16–19, 95%. The flow rate was 0.3 mL/min, and the injection volume was 1 µL. The use of reversed phase separation combined with the stationary phase biphenyl substituents promoted the appropriate separation of polar, mildly hydrophobic compounds with a focus on polyphenols and other functional phytometabolites part of specific families. Chromatographic stream was ionized via heated electrospray ionization (H-ESI) with a static spray voltage of −3.2 kV for negative and 3.5 kV for positive ions, while ion transfer tube and vaporizer temperature were set at 300 and 320 °C, respectively. Sheath gas flow and auxiliary gas flow were 50 and 10 arbitrary units, respectively. Analytes were scanned in polarity switching mode in the *m*/*z* range 120–1500 in full scan (FS) and data-dependent scanning mode (ddMS2), with mild trapping configuration and an expected chromatographic peak width of 7 s. The analyzer resolution was set at 120,000 (FWHM at *m*/*z* 200) for FS and data acquired in profile mode with a normalized AGC target of 100%. Separate methods for tandem MS experiments in ddMS2 were scheduled at the end of the batch through four overlapping acquisition segments (*m*/*z* 120–450, *m*/*z* 440–750, *m*/*z* 740–1050, and *m*/*z* 1040–1500). Within each experiment, a fragmentation spectrum was added to the chromatographic profile, isotopic pattern, and molecular formulas by combining positive (or negative) FS with ddMS2. For tandem MS acquisition, the settings were as follows: resolution of 60,000 (FWHM at *m*/*z* 200), isolation window of *m*/*z* 1.5 and 20, 45 and 60% normalized collision energy. Data profiles were acquired with an intensity threshold fixed at 50,000 (area counts); the dynamic exclusion was customized by considering a time window of 3.5 s and a mass tolerance within 5 ppm. A targeted mass exclusion list was manually annotated within the full scan range by injecting blank procedural samples and LC solvents. EASY-IC^®^, employing fluoranthene in both positive ion mode (*m*/*z* 202.0777 [M]^+^) and negative ion mode (*m*/*z* 202.0788 [M]^−^), was used to enhance mass centering and accuracy throughout the scan-to-scan acquisition. Profile data were collected using Xcalibur 4.5 (Thermo Fisher Scientific, Waltham, MA, USA); resolving power, response, and reproducibility of fragmentation spectra were checked by using Free Style software (v. 1.8, Thermo Fisher Scientific) during batch acquisition.

### 2.3. Bioinformatic Analysis of Untargeted Metabolomics Data

Raw files including procedural blank samples were loaded in Compound Discoverer v.3.3.1 (Thermo Fisher Scientific) and analyzed using an untargeted metabolomic workflow suitable for the annotation and identification of the compounds even at low abundance, with a minimum number of scan points of 8 and a mass tolerance within 3 ppm. Before matching pure analytical standards in the form of a mzVault library and publicly available database spectral matching (flavonoid database, Arita lab) [18], signals were detected and grouped according to their analytical behavior. Spectral features, i.e., putative molecular formula assignment through isotopic pattern and exact masses, formulas, and fragmentation patterns were matched with free databases, including mzCloud (https://www.mzcloud.org), ChemSpider (https://www.chemspider.com), Phenol-Explorer (https://phenol-explorer.eu), and Natural Products Atlas (https://www.npatlas.org). Signals were filtered upon removal of the background, compounds without MS2 fragments, as well as a number of scans below 8 [19]. Molecular network was built according to the chemical nature of each compound, focusing on similar biotransformation and common fragmentation spectrum. Compounds were grouped into a neural network, in which each node represented a molecule, the size was linked to compound area, whereas the node color indicated the spectral library (light green for mzCloud or mzVault, dark green for other databases such as ChemSpider). Finally, the identified compounds were quantified by using calibration curves built with authentic reference standards in the range 0 (solvent blank) to 10 mg/L according to Table S1.

### 2.4. Eukaryotic Cell Assays

#### 2.4.1. MTT Assay: Effect of *R. tomentosum* on Cell Viability

To evaluate *R. tomentosum* extract toxicity on eukaryotic cell lines, the 3-(4,5-dimethylthiazol-2-yl)-2,5-diphenyltetrazolium bromide (MTT) assay was performed on two immortalized and two cancer cell lines. HaCaT (immortalized human keratinocytes) were from Innoprot (Biscay, Derio, Spain); HeLa (human cervical cancer cells), Balb/c-3T3 (immortalized murine fibroblasts), and SVT2 (virus 40-transformed murine fibroblasts) were obtained from ATCC (Manassas, VA, USA). Cells were cultured as previously described [20]. The MTT assay was performed by plating cells in 96-well plates at the following density: 2 × 10^3^ cell/well for HaCaT and HeLa; 3 × 10^3^ cell/well for Balb/c-3T3; and 1.5 × 10^3^ cell/well for SVT2. Twenty-four hours after seeding, increasing concentrations of *R. tomentosum* extract (0.5–2%, *v*/*v*) were added to the cells for 48 h. At the end of the experiment, the MTT assay was performed [21]. Cell survival was expressed as the percentage of viable cells in the presence of the extract compared to the controls. Two groups of cells were used as controls, i.e., untreated cells and cells supplemented with the highest volume of hydroalcoholic buffer used (70% ethanol and 30% H_2_O, *v*/*v*).

#### 2.4.2. DCDFA Assay: Protective Effect of *R. tomentosum* Extract on HaCaT Cells

For the DCFDA assay, HaCaT cells were plated and incubated as described previously [20]. The extract concentration used was 0.5% (*v*/*v*).

### 2.5. Bacterial Cells Assay

#### 2.5.1. Antibacterial Activity of the *R. tomentosum* Extract

A broth microdilution assay was used to evaluate minimum inhibitory concentration (MIC). The following five plant-beneficial bacterial strains were used: *Pseudomonas fluorescens*, *Paenibacillus* sp., *Bacillus amyloliquefaciens*, *Rhodococcus qingshengii*, and *Bacillus velenzensis*; the following eight phytopathogens bacterial strains were tested: *Pseudomonas cichorii*, *Pseudomonas syringae*, *Curtobacterium flaccumfaciens*, *Agrobacterium tumefaciens*, *Clavibacter michiganensis* IPSP-001, *Clavibacter michiganensis* IPSP-002, *Xanthomonas vesicatoria*, and *Xanthomonas campestris*. All bacterial strains were obtained from the CNR-IPSP collection. A single colony of each bacterial strain was inoculated overnight at 28 °C in 5 mL of LB medium. Fifty µL of cultures of exponentially growing bacteria and increasing concentrations of *R. tomentosum* extract (up to 10%, *v*/*v*) were combined in a 96-well plate, with a final volume of 100 µL. Different antibiotics were used as positive controls as follows: ampicillin for *R. qingshengii*, *B. velenzensis,* and *X. vesicatoria*; streptomycin for *B. amyloliquefaciens*, *P. syringae*, *X. campestris,* and *A. tumefaciens*; kanamycin for *P. cichorii*; gentamycin for *P. fluorescens*, *Paenibacillus* sp.; tetracycline for *C. flaccumfaciens*, *C. michiganensis* IPSP-001, and *C. michiganensis* IPSP-002. After an overnight incubation at 28 °C, the optical density at 600 nm (O.D. 600 nm) of each mixture was measured using a multiplate reader GloMax Explorer (Promega, Madison, WI, USA). Samples were then plated on LB-agar and minimum bactericidal concentration (MBC) was evaluated after an overnight incubation at 28 °C.

#### 2.5.2. Effect of the *R. tomentosum* Extract on Bacterial Cell Proliferation

To evaluate bacterial cell proliferation, *R. tomentosum* extract was tested (0.5%, *v*/*v*) on the above-reported beneficial bacterial strains after incubation at 28 °C, for 24 h. O.D. at 600 nm was measured using a multiplate reader GloMax Explorer (Promega, Madison, WI, USA).

#### 2.5.3. Fungal Spore Germination Assay

Two phytopathogenic fungi, *Botrytis cinerea* and *Fusarium oxysporum* and one biocontrol agent, *Trichoderma atroviride* AGR2, were tested. All fungal strains were obtained from the CNR-IPSP collection. For each fungus, 10 μL of 1 × 10^4^ spore/mL spore suspension and 30 μL of potato dextrose broth (PDB) (Difco) were added in a 96 well-plate. *R. tomentosum* extract was tested up to 2.5% (*v*/*v*). In parallel, a hydroalcoholic solution (70%, *v*/*v*) was used as a buffer control. Volumes were adjusted (100 μL total volume) by adding distilled sterile water. Spore germination was analyzed after 20 h incubation using an Axiovert 5 digital inverted microscope (Zeiss, Oberkochen, Germany). The percentage of well coverage was quantified using Zeiss software (Labscope 4.2.1). Each well was divided into three regions, and the germination values obtained from the three regions were averaged. This procedure was repeated for each technical replicate.

### 2.6. Statistical Analysis

For all the experiments, each batch was tested in at least three independent analyses, each carried out in triplicate. Results are presented as the mean value of three independent experiments (mean ± S.D.) and were analyzed by one-way ANOVA followed by Bonferroni (post hoc) test, using GraphPad Prism for Windows, version 6.01 (Dotmatics, CA, USA).

## 3. Results

### 3.1. LC-MS/MS Analysis of the R. tomentosum Hydroalcoholic Extract

**Figure 1:**
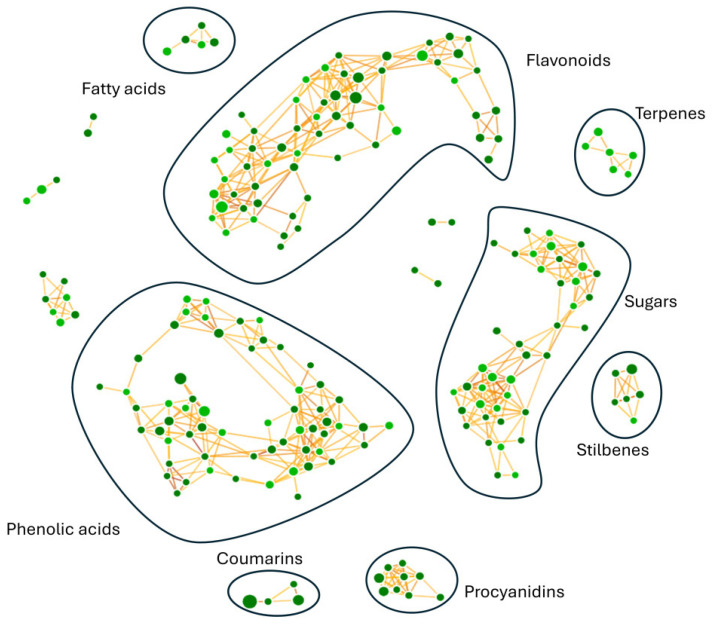
Molecular network obtained for *R. tomentosum* hydroalcoholic extract. Phenolic acids, coumarins, sugars, terpenes, stilbenes, flavonoids, procyanidins, and fatty acids were identified as molecular classes of interest. Phenolic acids and flavonoids were the most represented classes. Compounds were grouped into a neural network, in which each node represents a distinct molecule; the size is linked to compound area, whereas the node color indicates the spectral library used (light green for mzCloud or mzVault, dark green for other databases such as ChemSpider).

Untargeted metabolomic analysis provided a biochemical fingerprint of phytochemicals present in *R. tomentosum* hydroalcoholic extract. The optimization of the analytical workflow was the prerequisite for the annotation of chemical compounds of interest. Compound detection was carried out in negative polarity mode because of the chemical nature of the target analytes in the hydroalcoholic extract. Along with chromatographic separation and mass spectrometry configuration, the untargeted metabolomics workflow led to the annotation of 1526 compounds. Upon further processing, the number of metabolites was reduced to approximately 230 molecular species, as reported in Table S1, in which different levels of identification, accurate masses, chemical formulas, retention times, and spectra (diagnostic ions and reference fragments) are reported, in line with the metabolomics standard initiative [19]. Identified molecules were grouped based on their fragmentation patterns and predicted by biochemical transformations (glycosylation, methylation, hydration, dehydration, saturation, and oxidation) through a dedicated molecular network (Figure 1), highlighting compounds with a similarity score higher than 50%. Neural networks yielded several distinct clusters, with most of the molecules falling into flavonols, flavanols, hydroxybenzoic acid derivatives, sugars, hydroxycinnamic acid derivatives, terpenes, fatty acids, stilbenes, chalcones, and coumarins.

**Figure 2:**
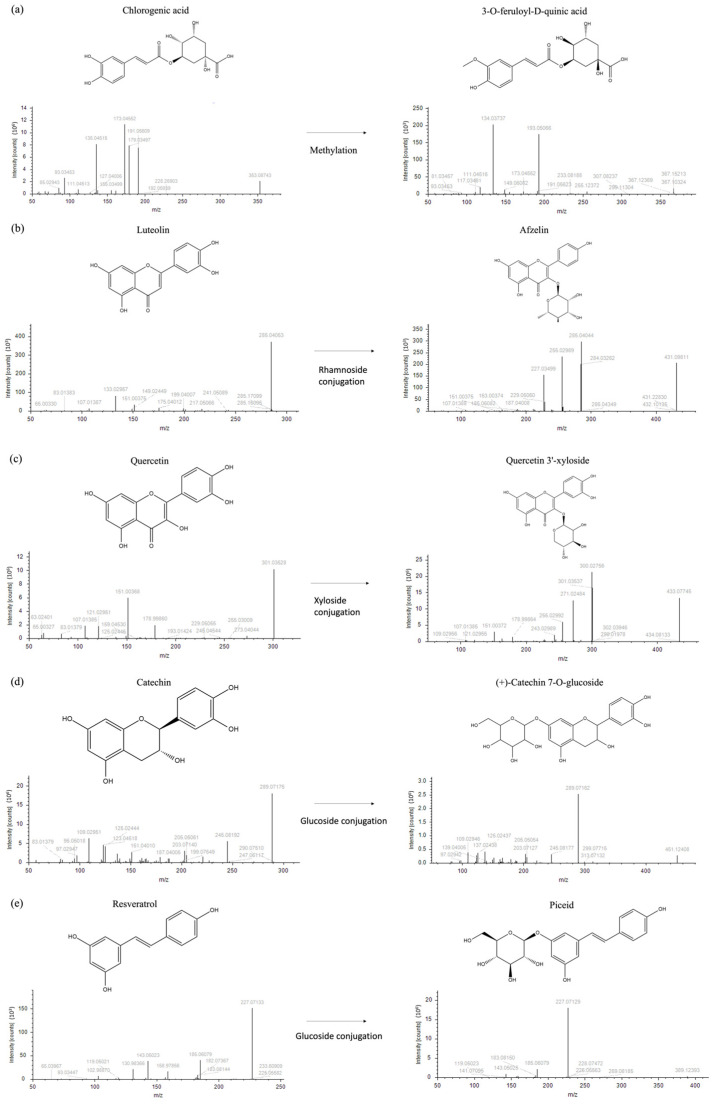

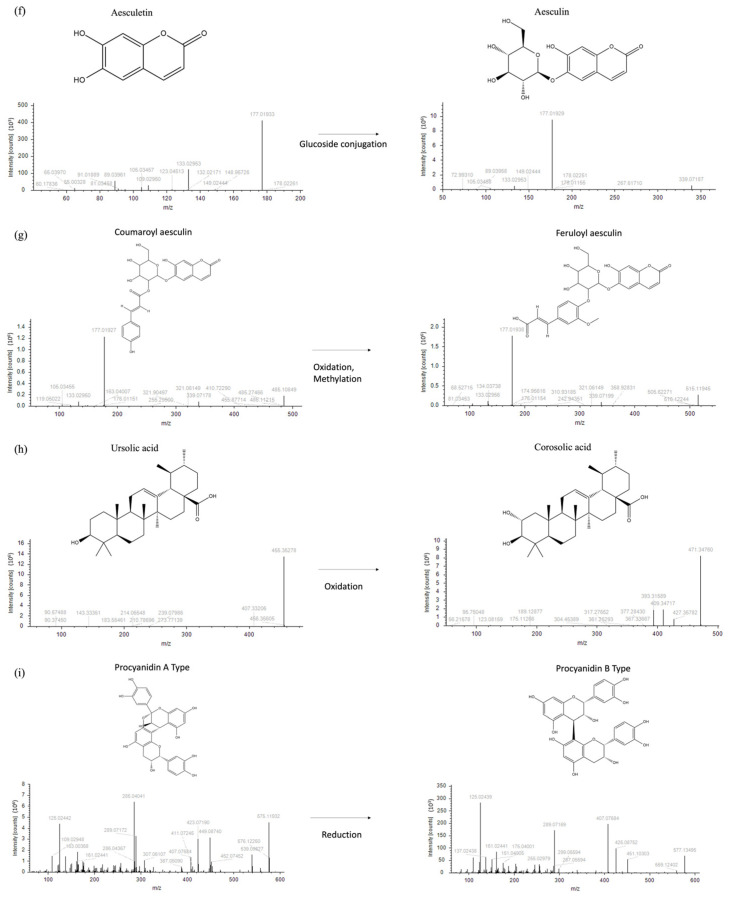
Fragmentation spectra and biochemical transformation of different compounds. (**a**) Methylation of chlorogenic acid to form 3-O-feruloyl-D-quinic acid; (**b**) conjugation of rhamnoside and luteolin to obtain afzelin; (**c**) conjugation of xyloside and quercetin to form quercetin 3′-xyloside; (**d**) conjugation of glucoside and catechin to obtain catechin 7-O-glucoside; (**e**) conjugation of glucoside and resveratrol to form piceid; (**f**) conjugation of glucoside and aesculetin to obtain aesculin; (**g**) oxidation and methylation of coumaroyl aesculin to form feruloyl aesculin; (**h**) oxidation of ursolic acid to obtain corosolic acid; and (**i**) procyanidin B type formation upon reduction of procyanidin A.

Each node is interjoined by different links, as a result of multiple iterative processes connecting compound fragmentation spectra and biochemical transformations typical of the chemical classes spotted in Figure 2. Such biochemical reactions represented the paradigm for the construction of the molecular network and provided the bases for compound grouping and their further characterization. Moving through phenolic acids, the methylation reaction linking fragmentation spectra of chlorogenic acid and 3-feruloyl-D-quinic was outlined (Figure 2a). Chlorogenic acid was characterized by the presence of the diagnostic fragment ions at *m*/*z* 173.045, 191.056, and 135.045, while 3-feruloyl-D-quinic acid exhibited the key ions at *m*/*z* 193.050 and 134.037. In Figure 2b–d, luteolin to afzelin, quercetin to quercetin 3′-xyloside, and catechin to catechin 7-*O*-glucoside, respectively, were described by the mean of enzymatic glycosylation reaction. Glycosylated compound spectra were characterized by an intense signal of fragment ions of the aglycone (*m*/*z* 285.040 for luteolin, 301.035 for quercetin, and 289.071 for catechin). In Figure 2e, glucoside conjunction of resveratrol into piceid was described. Both fragmentation spectra contained diagnostic fragment ions of resveratrol (*m*/*z* 227.071, 185.0607, and 143.0502), only differing for the presence of a hexose moiety (*m*/*z* 389.12393 and 227.071, respectively). In coumarin chemical classes, two examples of biotransformation were used to track molecular grouping. In Figure 2f,g, spectra reported the main fragment ions of aesculetin (*m*/*z* 177.019 and 133.029), which is the skeleton of all identified compounds. For triterpenoids, the oxidation of ursolic acid to corosolic acid was detailed (Figure 2h), emphasizing how compounds can be interconnected through oxidation reaction. Reduction in procyanidin A-type into procyanidin B-type was shown in Figure 2i. Procyanidin A type was characterized by typical fragment ions as *m*/*z* 285.040, 289.071, 125.024, 449.087, and 575.119, whereas procyanidin B type contained the following typical fragment ions: *m*/*z* 125.024, 289.071, 407.077, and 161.024.

**Figure 3:**
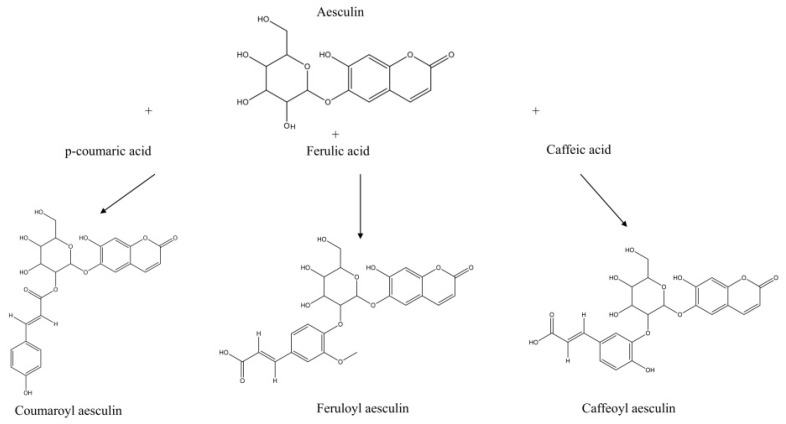
Aesculin derivatives. Aesculin derivatives (coumaroyl aesculin, feruloyl aesculin, and caffeoyl aesculin) and tentative assignments according to fragmentation spectra and molecular network with biotransformation and candidates screening. See Figure S1 for details on fragmentation spectra.

Besides the intrinsic number of annotated compounds within each chemical class, the molecular network provided information for compound assignment and identification through pure analytical standards. Table 1 reports the mass spectral identification of compounds based on their relative abundance (only compounds with a relative abundance > 0.5% are reported). Interestingly, coumarins were the most represented class of molecules identified in the extract (24%), followed by flavonols (20%), flavanols (11%), hydroxybenzoic acid derivatives and sugars (9%), hydroxycinnamic acid derivatives and terpenes (6%), fatty acids (5%), stilbenes (3%), and chalcones (1%). Focusing on coumarins as the most representative chemical class, several compounds were annotated, and their identity was tentatively confirmed through fragmentation spectra and accurate masses. Different derivatives (coumaroyl aesculin, feruloyl aesculin, and caffeoyl aesculin) were tentatively identified here for the first time based on typical aesculin fragments ions (*m*/*z* 177.01929, 176.01155, and 339.07190). These molecules were characterized by a hydroxycinnamic acid derivative (p-coumaric acid, ferulic acid, and caffeic acid, respectively) O-linked to aesculin through the glycoside moiety, as shown in Figure 3 and Figure S1, with the latter ones reporting the corresponding tandem mass spectra. As reported in Table S1, two additional unknown aesculin derivatives were detected in the hydroalcoholic extract; their tentative molecular assignment was based on the observation of fragment ions that were identical to those of aesculin and its aglycone form.

The relative concentration (mg/L) of the identified compounds in the hydroalcoholic extract was obtained using pure chemical standards for different chemical classes (Table 1 and Table S1). Catechin, luteolin, and naringenin were used as standards for flavonoids, whereas 3,5-dihydroxybenzoic acid, caffeic acid, and chlorogenic acid were utilized for phenolic acids; 3,3′,4′,5-tetrahydroxystilbene was used as a standard compound for stilbenes, while asiatic acid, agnuside, and abscisic acid were utilized for terpenes and derivatives.

### 3.2. Biological Activity

#### 3.2.1. Effect of *R. tomentosum* Hydroalcoholic Extract on Eukaryotic Cell Viability

**Figure 4:**
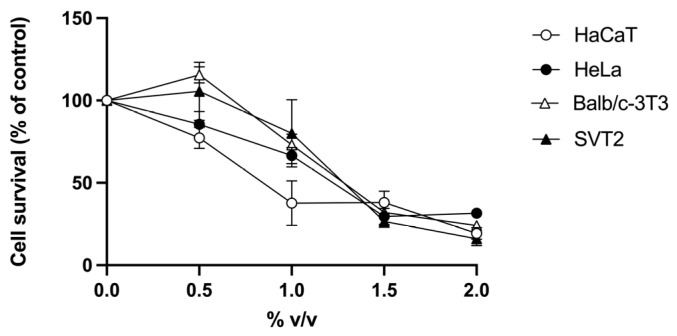
Effect of *R. tomentosum* extract on cell viability. HaCaT (white circles), HeLa (black circles), Balb/c-3T3 (white triangles), and SVT2 (black triangles) cells were incubated with increasing concentrations of the extract (0.5–2%, *v*/*v*) for 48 h. Cell survival is reported as a function of the extract tested. Data shown are means ± S.D. of three independent experiments.

*R. tomentosum* hydroalcoholic extract was tested on two immortalized (human HaCaT keratinocytes and murine embryonic Balb/c-3T3 fibroblasts) and two cancer (human cervical HeLa cancer cells and virus 40-transformed murine SVT2 embryonic fibroblasts) cell lines. Cells were incubated with increasing concentrations of *R. tomentosum* extract (0.5–2%, *v*/*v*), and cell viability was evaluated by the MTT assay. As shown in Figure 4, a similar trend was observed for all the cell lines tested, with HaCaT being the most sensitive cells. From the MTT assay results, IC_50_ values were calculated (% *v*/*v*, Table 2). This parameter indicates the concentration of the extract causing 50% cell death and is related to the sensitivity of cells to the extract.

#### 3.2.2. Protective Effect of *R. tomentosum* Hydroalcoholic Extract on UVA-Stressed HaCaT Cells

**Figure 5:**
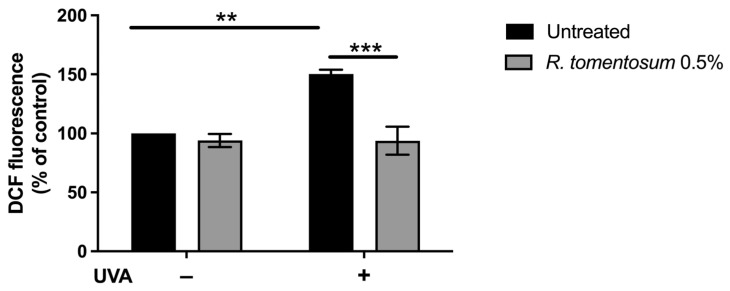
Antioxidant activity of *R. tomentosum* hydroalcoholic extract determined by DCFDA assay. HaCaT cells were incubated in the presence of 0.5% (*v*/*v*) of the extract for 2 h, stressed with a UVA lamp (100 J/cm^2^), and intracellular ROS level was measured. ROS production was expressed as a percentage of the intensity of DCF fluorescence compared to untreated cells. Black bars refer to control cells, and gray bars refer to cells incubated with the extract, in the absence (−) or presence (+) of UVA stress. Data shown are mean values ± S.D. of three independent experiments. ** indicates *p* < 0.01, and *** indicates *p* < 0.001. The lines above the bars indicate the two samples compared for statistical analysis.

Hydroalcoholic plant extracts contain antioxidant molecules that can find application in different fields [22,23,24]. Thus, the possible protective effect of *R. tomentosum* extract against oxidative stress was tested using HaCaT cells, as keratinocytes are normally exposed to external stimuli, such as sun UVA radiation. To this purpose, 0.5% (*v*/*v*) hydroalcoholic extract was chosen for the analysis, as it represents the highest concentration without toxic effect on HaCaT cells (MTT assay). Cells were incubated with the extract for 2 h, and then stressed by an UVA lamp, typically used in the nail industry [25]. H_2_-DCFDA probe was added, and intracellular ROS levels were measured. As shown in Figure 5, upon UVA stress, control cells showed a significant increase in ROS levels with respect to non-stressed cells (150%, black bars, *p* < 0.01). When cells were incubated with *R. tomentosum* extract and then exposed to UVA radiations, a significant inhibition in ROS production was observed (93%, *p* < 0.001). No alteration in ROS levels was observed when HaCaT cells were incubated in the presence of the extract but in the absence of irradiation.

#### 3.2.3. Evaluation of the Antibacterial Activity of *R. tomentosum* Hydroalcoholic Extract

Many of the molecules tentatively identified in *R. tomentosum* extract are related to antibacterial activity often associated with the activity of these compounds against human pathogenic bacteria and not on phytopathogens [26,27,28]. Thus, to further explore the biological activity of the extract, its potential antibacterial properties towards different beneficial and pathogenic phytobacteria were tested via broth microdilution assay. After an overnight bacterial inoculum, cells were plated in a 96-well plate in the presence of increasing concentrations of the hydroalcoholic extract (1–10%, *v*/*v*), and MIC values were evaluated by spectrophotometric measurements. As shown in Table 3, *R. tomentosum* extract inhibited the growth of two phytopathogens, i.e., C. *michiganensis* IPSP-001 and *C. michiganensis* IPSP-002, and of the beneficial *B. velenzensis* strain. The same MIC values (10%, *v*/*v*) were measured for *C. michiganensis* IPSP-002 and *B. velenzensis*, whereas an MIC value of 2.5% (*v*/*v*) was obtained for *C. michiganensis* IPSP-001. The MBC values of the *R. tomentosum* extract were determined by transferring samples from the broths utilized for MIC determination onto solid medium. The measured MBC values were higher than the maximum concentration tested (10%, *v*/*v*) for all bacterial strains analyzed (Table 3), suggesting a bacteriostatic but not bactericidal effect on *C. michiganensis* IPSP-001, *C. michiganensis* IPSP-002, and *B. velenzensis*.

#### 3.2.4. Effect of the *R. tomentosum* Hydroalcoholic Extract on Bacterial Cell Proliferation

Once proved that the extract did not show any inhibitory effect on beneficial bacterial strains, with the exception of *B. velenzensis*, other beneficial bacterial strains were incubated with 0.5% (*v*/*v*) *R. tomentosum* extract to evaluate its possible use as a bacterial growth stimulant. This concentration was selected because of its biocompatibility (Figure 4) and its antioxidant activity (Figure 5) on eukaryotic cells. Results showed that the *R. tomentosum* extract notably stimulated *B. amyloliquefaciens* growth by 42% after 24 h incubation, whereas it had no effect on the other tested bacterial strains.

#### 3.2.5. Effect of *R. tomentosum* Hydroalcoholic Extract on Fungal Spore Germination

**Figure 6:**
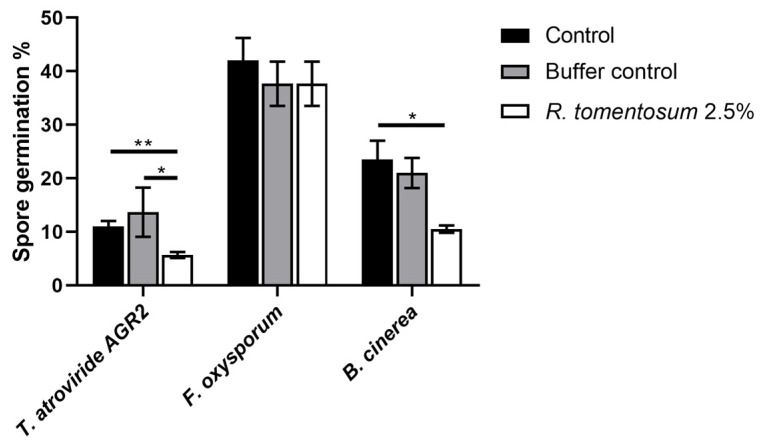
Effect of *R. tomentosum* hydroalcoholic extract on spore germination. *T. atroviride* AGR2, *F. oxysporum,* and *B. cinerea* spore germination after 20 h of incubation in presence of the *R. tomentosum* hydroalcoholic extract. A total of 2.5% (*v*/*v*) of the extract and the hydroalcoholic buffer were used. Microscopic analyses were performed using an inverted microscope Zeiss Axiovert 5 digital. Data shown are mean values ± S.D. of three independent experiments. * indicates *p* < 0.05; ** indicates *p* < 0.01.

The *R. tomentosum* extract was tested on the spores of the following three fungi: *Trichoderma atroviride* AGR2, *Fusarium oxysporum,* and *Botrytis cinerea*. *T. atroviride* AGR2 is a beneficial fungus, whereas *F. oxysporum* and *B. cinerea* are phytopathogens. Spore germination was measured after 20 h of incubation in the presence or absence of the extract (up to 2.5%, *v*/*v*), as described in Section 2. The antifungal effect was observed when spores were incubated with 2.5% (*v*/*v*) of the extract, as reported in Figure 6. The hydroalcoholic buffer (without the extract) was tested at the same concentration (2.5%, *v*/*v*) and did not show any significant toxicity on the three analyzed fungi. Surprisingly, *T. atroviride* AGR2 and *B. cinerea* spore germination was significantly affected by *R. tomentosum* extract (about 50% inhibition), whereas no effect on *F. oxysporum* spore germination was observed in the presence of the extract.

## 4. Discussion

The aromatic plant *R. tomentosum* represents a sustainable source of bioactive compounds with several applications in multiple fields. In this study, a mass spectrometry characterization of a commercially available *R. tomentosum* hydroalcoholic extract tackled its chemical composition, providing a molecular fingerprinting of the extract. According to the main results reported for other *Rhododendron* species, flavanols, flavonols, hydroxybenzoic acids, hydroxycinnamic acids, coumarins, stillbenes, fatty acids, sugars, organic acids, and terpens represented the main constituents. In line with the chemical profile here summarized, afzelin, luteolin, phloretin (flavonoids), dihydromyricetin (flavanonols), 1-O-vanilloyl-beta-D-glucose (glucosides), sinapinate (alkaloids), 3-p-coumaroylquinic acid (hydroxynnamic acids), scopoline (coumarins), (10E)-9,13-dihydroxy-10-octadecenoic acid (fatty acids), and asiatic acid (terpenoids) were outlined as key biomarkers in *R. adamsii*, *R. lapponicum*, and *R. burjaticum* [29]. Other flavonoids, such as quercetin 3-(6″-p-hydroxybenzoylgalactoside), naringenin, and gallocatechin were detected in *R. dauricum* (leaves), *R. hainanense* (aerial parts), *R. molle* (fruits) [16], whereas in quercetin 3′-xyloside response paralleled the one reported in *R. sichotense* leaves and stems [30]. Similarly, the concentrations of quercetin and its derivatives, catechin, procyanidins, and chlorogenic acid, were at the same order of magnitude as reported in *R. groenlandicum* and *tomentosum* [31]. In *R. tomentosum* leaves and twigs, diosmetin, procyanidins (flavonoids), and ellagic acid (catechols) were identified as the main compounds upon supercritical CO_2_ extraction [32]. In the present study, several phytochemicals belonging to flavonoids, such as luteolin, 3-methoxyluteolin, and newly reported aesculin derivatives (coumarins), were identified, for the first time in *R. tomentosum* tips of twigs, thus expanding its annotated chemical profile. To the best of our knowledge, luteolin has been detected only in *R. luteum* sweet leaf extracts, at low concentration [33]. Of note, coumaroyl-, feruloyl-, and caffeoyl aesculin (aesculin-hydroxycinnamic acid derivatives) were identified for the first time in this study, highlighting the possibility that the glycoside moiety of aesculin might be the target for biochemical processes leading to the formation of coumaroyl aesculin, feruloyl aesculin, and caffeoyl aesculin as part of the metabolites arising from the interplay between p-coumaroyl-coenzyme A and UDP-glucose-dependent glucosyltransferases [34].

Flavonoids and coumarins are well-known antioxidants, which act by different mechanisms of action [35]. Noteworthy, this is the first report on the protective effect of *R. tomentosum* hydroalcoholic extract against UVA-induced oxidative stress on a cell-based model. Previously, *R. tomentosum* and *R. przewalskii* extracts were successfully tested only by in vitro analyses, with *R. przewalskii* extract effective also on RAW 264.7 cells, and the antioxidant activity mainly attributed to the presence of flavonoids [17,36]. Additionally, quercetin and luteolin exhibit anti-inflammatory and cytoprotective activities through modulation of NF-κB, MAPK, and COX/LOX pathways, as well as activation of the Nrf-2/Keap1 pathway [37], mechanisms involved in different cell-defense response. In *R. tomentosum* hydroalcoholic extract, coumarins are the most represented metabolites, and their abundance likely contributes to several of the biological effects described in this study. Molecules belonging to coumarins, such as scopoletin, aesculin, and fraxetin, are widely recognized for their antioxidant, antimicrobial, and immunomodulatory properties [38]. Beyond direct biological activity, coumarins have emerged as key mediators of plant–microbe interactions. Recent studies have demonstrated that they shape the rhizosphere microbiome by selectively inhibiting pathogens while promoting beneficial taxa, including *Pseudomonas* and *Bacillus* species, due to their redox chemistry and metal-mobilizing capacity [39,40]. Recent work expanded their ecological role, showing that coumarins produced by pepper roots can restructure the soil microbiome to enhance pesticide-degrading bacteria, and simultaneously improve fruit nutritional quality [41]. This evidence reinforces the idea that coumarins are key modulators of microbial chemical signals, promoting plant protection and detoxification, in line with our results. Indeed, coumarins impair microbial quorum sensing, inhibit essential enzymes, and destabilize fungal cell walls [38]. These mechanistic insights provide a plausible explanation for the moderate bacteriostatic effects observed against *C. michiganensis* and the suppression of spore germination in *B. cinerea* and *T. atroviride* AGR2. Moreover, *R. tomentosum* extract stimulated the growth of the beneficial *B. amyloliquefaciens*, in agreement with Wang and coworkers, who ascribed the dual behavior to the presence of coumarins in *R. tomentosum* extract [41]. Such activities are particularly relevant for agricultural applications, where the ability to favor plant-beneficial bacteria while inhibiting pathogens is highly desirable. Moreover, these findings suggest a potential role for such extracts as supportive tools for modern agriculture.

**Figure 7:**
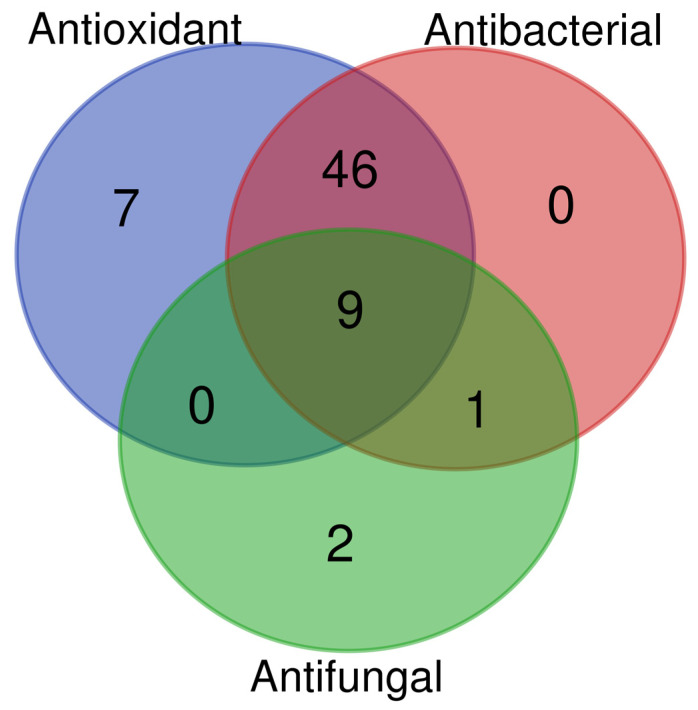
Venn diagram generated from the literature data analysis. Metabolites were classified based on their antioxidant, antibacterial, and antifungal activities.

In Figure 7, the identified metabolites were clustered according to their antioxidant, antibacterial, and antifungal properties, based on functional data available in the literature (https://bioinformatics.psb.ugent.be/webtools/Venn/, accessed on 3 November 2025). Most of the metabolites showed antioxidant and antibacterial activities, while a small subset of the identified metabolites (nine), including caffeic acid [42], gallic acid [43], quercetin [28,44], catechin, and scopoletin [26], exhibited all three activities.

## 5. Conclusions

This study recognizes *R. tomentosum* as a source of bioactive compounds, emphasizing the critical need to connect chemical composition to functional properties in phytochemical research. Reporting the first-ever tentative identification of novel aesculin derivatives, this paper extends the phytochemical profile previously attributed to this species, presenting new insights into *R. tomentosum* biochemical signature. By integrating metabolomic profile with assays evaluating the antioxidant, antibacterial, and antifungal potential, this work provides a framework for bridging the chemical composition of the plant extract to its functional properties. The proposed approach highlights chemical and biological information and establishes a foundation for their potential application in sustainable agriculture.

## Figures and Tables

**Table 1:** LC-MS/MS spectral analysis of phytochemicals present in *R. tomentosum* hydroalcoholic extract. Tentatively identified species are reported, together with their formula, calculated M.W., *m*/*z* ratio, retention time, abundance, and relative concentration. The relative abundance for each class of molecules refers to the samples reported in Table S1.

Compound	Formula	Calculated M.W.	*m*/*z*	RT (min)	Relative Abundance (%)	Standard	Average Concentration (mg/L)	S.D.
Flavanols					10.97			
Catechin	C_15_H_14_O_6_	290.0787	289.07143	3.291	4.09	Catechin	130.007	0.561
(+)-Procyanidin A type	C_30_H_24_O_12_	576.12629	575.11899	5.116	2.06	Catechin	65.077	0.666
(+)-Procyanidin A type isomer I	C_30_H_24_O_12_	576.1264	575.11911	5.583	1.76	Catechin	55.688	0.460
Epicatechin	C_15_H_14_O_6_	290.07882	289.07155	3.754	1.20	Catechin	37.856	0.347
(+)-Catechin 7-O-glucoside	C_21_H_24_O_11_	452.13142	451.12413	2.218	0.69	Catechin	21.508	0.464
Flavonols					20.14			
Quercetin 3′-xyloside	C_20_H_18_O_11_	434.08432	433.07701	5.311	4.50	Luteolin	11.272	0.090
Quercetin 3-(6″-*p*-hydroxybenzoylgalactoside)	C_28_H_24_O_14_	584.11602	583.10873	6.244	3.00	Luteolin	7.495	0.091
Isoquercetin	C_21_H_20_O_12_	464.09494	463.08765	4.71	2.98	Luteolin	7.415	0.145
Quercitrin	C_21_H_20_O_11_	448.1001	447.0928	5.482	2.46	Luteolin	6.158	0.048
Quercetin coumaroyl hexoside	C_30_H_26_O_14_	610.13191	609.12463	7.254	1.69	Luteolin	4.250	0.028
Quercetin acetyl hexoside	C_23_H_22_O_13_	506.10562	505.09834	6.196	1.39	Luteolin	3.492	0.019
Quercetin	C_15_H_10_O_7_	302.0425	301.03522	7.555	1.09	Luteolin	2.704	0.041
Quercetin 3′-xyloside isomer	C_20_H_18_O_11_	434.08452	433.07724	5.091	0.85	Luteolin	2.127	0.021
Hydroxybenzoic acid and derivatives					9.31			
Homovanillic acid	C_9_H_10_O_4_	182.05777	181.05049	5.699	1.26	3,5-Dihydroxybenzoic acid	36.258	0.693
Protocatechuic aldehyde	C_7_H_6_O_3_	138.03157	137.02429	2.994	1.22	3,5-Dihydroxybenzoic acid	35.227	0.435
1-O-vanilloyl-beta-D-glucose	C_14_H_18_O_9_	330.09483	329.08756	3.832	0.99	3,5-Dihydroxybenzoic acid	28.572	0.338
1-O-vanilloyl-beta-D-glucose isomer	C_14_H_18_O_9_	330.09475	329.08751	3.244	0.92	3,5-Dihydroxybenzoic acid	26.547	0.206
3,5-Dihydroxybenzoic acid	C_7_H_6_O_4_	154.02647	153.0192	1.928	0.90	3,5-Dihydroxybenzoic acid	25.969	0.416
Dihydroxybenzoic acid hexoside	C_13_H_16_O_9_	316.07918	315.0719	1.545	0.65	3,5-Dihydroxybenzoic acid	18.940	0.123
Vanillyl hexoside	C_14_H_20_O_8_	316.11558	315.10832	1.779	0.58	3,5-Dihydroxybenzoic acid	16.821	0.204
Salicylic Acid hexoside	C_13_H_16_O_8_	300.08432	299.07706	1.832	0.53	3,5-Dihydroxybenzoic acid	15.159	0.262
Hydroxycinnamic acids and derivatives					5.82			
1-Caffeoylquinic acid	C_15_H_16_O_7_	308.08899	353.08719	2.449	2.24	Chlorogenic acid	171.559	1.760
Chlorogenic acid	C_16_H_18_O_9_	354.09444	353.08714	3.225	1.51	Chlorogenic acid	116.202	0.198
Coumarins					24.19			
Fraxin	C_16_H_18_O_10_	370.08925	369.08197	3.742	9.07			
Aesculin	C_15_H_16_O_9_	340.07881	339.07152	3.136	4.19			
Fraxetin	C_10_H_8_O_5_	208.03694	207.02966	4.264	3.08			
5,7-Dihydroxychromone	C_9_H_6_O_4_	178.0264	177.01912	3.59	2.99			
Feruloyl aesculin	C_25_H_24_O_12_	516.12657	515.11929	6.878	0.73			
Scopolin	C_16_H_18_O_9_	354.09452	399.0927	3.647	0.71			
Aesuletin derivative	C_21_H_24_O_13_	484.12131	483.11403	4.124	0.60			
Coumaroyl aesculin	C_24_H_22_O_11_	486.116	485.10872	6.599	0.60			
3-acetyl-7-methoxychromen-2-one	C_12_H_10_O_4_	218.05775	217.05048	4.319	0.60			
Stilbenes					3.24			
Piceid	C_20_H_22_O_8_	390.13097	435.12915	4.91	2.65	3,3′,4′,5-Tetrahydroxystilbene	18.207	0.255
Fatty acids					4.61			
10,16-Dihydroxyhexadecanoic acid	C_16_H_32_O_4_	288.22996	287.22269	9.143	0.77			
(10E)-9,13-Dihydroxy-10-octadecenoic acid	C_18_H_34_O_4_	314.2455	313.23822	12.379	0.76			
Threonic acid, L-	C_4_H_8_O_5_	136.03703	135.02975	0.709	0.69			
(10E,12E)-9-Hydroxy-10,12-octadecadienoic acid	C_18_H_32_O_3_	296.23501	295.22774	14.002	0.50			
Sugars and derivatives					8.66			
Benzyl beta-D-primeveroside	C_18_H_26_O_10_	402.15201	447.15021	3.344	1.05			
Salidroside	C_14_H_20_O_7_	300.12032	345.11847	1.695	1.03			
Phenethyl beta-D-primeveroside	C_19_H_28_O_10_	416.16779	461.16597	3.976	1.02			
D-(−)-Arabinose	C_5_H_10_O_5_	150.05261	195.05081	0.7	0.90			
D-(+)-Glucose	C_6_H_12_O_6_	180.06318	179.05592	0.701	0.84			
Organic acids					1.9			
Arabic acid	C_5_H_10_O_6_	166.04756	165.04028	0.699	1.01			
Citric acid	C_6_H_8_O_7_	192.02681	191.01954	0.855	0.79			
Terpens and derivatives					6.21			
20S,24S-dihydroxydammer-25-en-3-one	C_30_H_50_O_3_	458.3755	503.37369	16.552	1.54	Asiatic acid	52.554	0.522
Nepetaside	C_16_H_26_O_8_	346.16222	345.15492	3.49	1.22	Agnuside	5.628	0.071
Ursolic acid	C_30_H_48_O_3_	456.35998	455.35269	15.681	0.90	Asiatic acid	30.746	0.496
Chalcones					1.31			
Neobavachalcone	C_17_H_14_O_5_	298.08389	297.07661	13.299	1.29			

**Table 2:** IC_50_ values (% *v*/*v*) of *R. tomentosum* hydroalcoholic extract on eukaryotic cells. Data shown are mean values ± S.D. of three independent experiments.

HaCaT	HeLa	Balb/c-3T3	SVT2
0.79 ± 0.01	1.23 ± 0.06	1.30 ± 0.04	1.30 ± 0.20

**Table 3:** Minimum inhibitory concentration (MIC, % *v*/*v*) and minimal bactericide concentration (MBC, % *v*/*v*). Values were determined for *R. tomentosum* hydroalcoholic extract tested on a panel of beneficial and phytopathogen bacterial strains. The following different antibiotics were used as positive control: ampicillin (^a^), streptomycin (^s^), kanamycin (^k^), gentamycin (^g^), and tetracycline (^t^). Values were obtained from a minimum of three independent experiments.

	*R. tomentosum* Extract (% *v*/*v*)		Positive Control (mg/mL)	
	MIC	MBC	MIC	MBC
Beneficial strains				
*B. velenzensis*	10	>10	0.55 ^a^	>1 ^a^
*Paenibacillus* sp.	>10	>10	0.5 ^g^	1 ^g^
*P. fluorescens*	>10	>10	0.5 ^g^	0.5 ^g^
*R. qingshengii*	>10	>10	1 ^a^	>1 ^a^
*B. amyloliquefaciens*	>10	>10	0.5 ^s^	>0.5 ^s^
Phytopathogen strains				
*P. syringae*	>10	>10	0.25 ^s^	0.5 ^s^
*P. cichorii*	>10	>10	0.01 ^k^	>0.01 ^k^
*C. michiganensis* IPSP-001	2.5	>10	0.03 ^t^	0.125 ^t^
*C. michiganensis* IPSP-002	10	>10	0.03 ^t^	0.125 ^t^
*C. flaccumfaciens*	>10	>10	0.125 ^t^	0.25 ^t^
*X. campestris*	>10	>10	1 ^s^	>1 ^s^
*X. vesicatoria*	>10	>10	0.25 ^a^	>1 ^a^
*A. tumefaciens*	>10	>10	1 ^s^	>1 ^s^

## Data Availability

All data generated during this study are included in this published article and its Supplementary information file.

## Supplementary Materials

The following supporting information can be downloaded at https://www.mdpi.com/article/10.3390/biom16010110/s1, Table S1: Tentatively identified species in the *R. tomentosum* hydroalcoholic extract determined by LC-MS/MS and bioinformatic analysis; Figure S1: Tandem mass spectra of caffeoyl aesculin, coumaroyl aesculin, and feruloyl aesculin.

## Abbreviations

The following abbreviations are used in this manuscript:

LC-MS/MS	Liquid Chromatography high-resolution Tandem Mass Spectrometry
MTT	3-(4,5- dimethylthiazol-2-yl)-2,5-diphenyltetrazolium bromide
DCFDA	DiChlorodihydroFluorescein DiAcetate
M.W.	Molecular Weight
IC_50_	Half-maximal Inhibitory Concentration
UVA	UltraViolet A
ROS	Reactive Oxygen Species
DCF	2′,7′-Dichlorofluorescein
MIC	Minimum Inhibitory Concentration
MBC	Minimum Bactericidal Concentration
ATCC	American Type Culture Collection
O.D.	Optical Density
CNR-IPSP	National Research Council-Institute for Sustainable Plant Protection
