# Supplementary material for: Insights into Bioactive Molecules in Rhododendron tomentosum: From Metabolomics to Biological Applications
Source: Biomolecules. 2026 Jan 8;16(1):110. doi: 10.3390/biom16010110 (PMC12839003; doi:10.3390/biom16010110)
Supplement: Supplementary file 1 [file biomolecules-16-00110-s001.zip › biomolecules-4039022-supplementary.pdf]

Insights into bioactive molecules in *Rhododendron tomentosum*: from metabolomics to biological applications

Giovanna Schiavone[1,2,†], Paola Imbimbo[1,†], Sabrina De Pascale [3], Rosalia Ferracane[3], Simonetta Caira[3], Andrea Scaloni[3], Antonio Dario Troise[3*], Daria Maria Monti[1*], Vincenzo Rocco[4], Daniela D'Esposito[2], Maurilia Maria Monti[2]

[1]Department of Chemical Sciences, University of Naples Federico II, Complesso Universitario Monte Sant'Angelo, via Cinthia 4, 80126 Naples, Italy;

[2]Institute for Sustainable Plant Protection, National Research Council, p.le E. Fermi 1, 80055 Portici, Italy;

[3]Institute for the Animal Production System in the Mediterranean Environment, National Research Council, p.le E. Fermi 1, 80055 Portici, Italy;

[4]CeMON S.r.l. Centro di Medicina Omeopatica Napoletano, viale Gramsci 18, 80122 Naples, Italy.

SUPPLEMENTARY INFORMATION

TABLE S1 Tentatively identified species in the *R. tomentosum* hydroalcoholic extract as determined by LC-MS/MS and bioinformatic analysis. Results include molecular annotation, chemical formulas, calculated M.W., *m/z* ratio, retention time, abundance and relative concentration.

Compound	Formula	Annot. DeltaMass [ppm]	Calculated M.W.	*m/z*	RT [min]	Relative abundance (%)	Standard	Average concentration (mg/L)	S.D.
**Flavanols**									
Catechin	$C_{15}H_{14}O_6$	-1.15	290.0787	289.07143	3.291	4.09	Catechin	130.007	0.561
(+)-Procyanidin A type	$C_{30}H_{24}O_{12}$	-0.84	576.12629	575.11899	5.116	2.06	Catechin	65.077	0.666
(+)-Procyanidin A type isomer I	$C_{30}H_{24}O_{12}$	-0.65	576.1264	575.11911	5.583	1.76	Catechin	55.688	0.460
Epicatechin	$C_{15}H_{14}O_6$	-0.74	290.07882	289.07155	3.754	1.20	Catechin	37.856	0.347
(+)-Catechin 7-O-glucoside	$C_{21}H_{24}O_{11}$	-0.97	452.13142	451.12413	2.218	0.69	Catechin	21.508	0.464
(+)-Procyanidin A type isomer II	$C_{30}H_{24}O_{12}$	-0.45	576.12652	575.1192	5.242	0.33	Catechin	10.565	0.081
(+)-Procyanidin B type	$C_{30}H_{26}O_{12}$	-0.72	578.14201	577.13474	3.435	0.22	Catechin	6.900	0.013
(+)-Procyanidin B type isomer I	$C_{30}H_{26}O_{12}$	-0.52	578.14213	577.13486	3.691	0.19	Catechin	5.977	0.098
Epigallocatechin 3-O-*p*-coumarate	$C_{24}H_{20}O_9$	-0.39	452.11056	451.10327	6.061	0.19	Epigallocatechin	3.705	0.096
(+)-Procyanidin A type isomer III	$C_{30}H_{24}O_{12}$	-0.06	576.12674	575.11945	5.742	0.10	Catechin	3.215	0.050
(+)-Procyanidin B type isomer II	$C_{30}H_{26}O_{12}$	-0.66	578.14204	577.13477	3.186	0.08	Catechin	2.570	0.039
(+)-Procyanidin A type isomer VI	$C_{30}H_{24}O_{12}$	-0.36	576.12657	575.11929	6.264	0.05	Catechin	1.592	0.059
(+)-Gallocatechin	$C_{15}H_{14}O_7$	0.07	306.07397	305.0667	2.103	0.01	Epigallocatechin	0.220	0.004
**Flavonols**									
Quercetin 3'-xyloside	$C_{20}H_{18}O_{11}$	-1.37	434.08432	433.07701	5.311	4.50	Luteolin	11.272	0.090
Quercetin 3-(6''-*p*-hydroxybenzoylgalactoside)	$C_{28}H_{24}O_{14}$	-1	584.11602	583.10873	6.244	3.00	Luteolin	7.495	0.091

Isoquercetin	$C_{21}H_{20}O_{12}$	-1.15	464.09494	463.08765	4.71	2.98	Luteolin	7.415	0.145
Quercitrin	$C_{21}H_{20}O_{11}$	-1.03	448.1001	447.0928	5.482	2.46	Luteolin	6.158	0.048
Quercetin coumaroy hexoside	$C_{30}H_{26}O_{14}$	-0.56	610.13191	609.12463	7.254	1.69	Luteolin	4.250	0.028
Quercetin acetyl hexoside	$C_{23}H_{22}O_{13}$	-0.82	506.10562	505.09834	6.196	1.39	Luteolin	3.492	0.019
Quercetin	$C_{15}H_{10}O_{7}$	-0.52	302.0425	301.03522	7.555	1.09	Luteolin	2.704	0.041
Quercetin 3'-xyloside	$C_{20}H_{18}O_{11}$	-0.9	434.08452	433.07724	5.091	0.85	Luteolin	2.127	0.021
Taxifolin 7- glucoside	$C_{21}H_{22}O_{12}$	-0.84	466.11073	465.10345	4.023	0.60	Luteolin	1.503	0.018
Quercetin ferulyl hexoside	$C_{31}H_{28}O_{15}$	-0.16	640.14271	639.13544	7.496	0.45	Luteolin	1.136	0.002
Quercetin 3-O-neohesperidoside	$C_{27}H_{30}O_{16}$	-0.18	610.15328	609.146	4.458	0.27	Luteolin	0.676	0.016
Taxifolin	$C_{15}H_{12}O_{7}$	-0.71	304.05809	303.05081	5.045	0.23	Luteolin	0.577	0.006
Myricitin pentoside	$C_{20}H_{18}O_{12}$	-0.83	450.07945	449.07217	5.594	0.23	Luteolin	0.564	0.005
3,7-Dimethylquercetin	$C_{17}H_{14}O_{7}$	-0.02	330.07394	329.06667	11.389	0.18	Luteolin	0.434	0.018
Myricetin 5-methyl ether	$C_{16}H_{12}O_{8}$	0.47	332.05337	331.0461	6.718	0.13	Luteolin	0.324	0.010
Peltatoside	$C_{26}H_{28}O_{16}$	-0.36	596.13752	595.13025	4.228	0.13	Luteolin	0.328	0.001
6-Methoxytaxifolin	$C_{16}H_{14}O_{8}$	-0.52	334.06869	333.06142	3.836	0.12	Luteolin	0.294	0.002
Quercetin derivative	$C_{27}H_{30}O_{14}$	0.15	578.16364	577.15636	6.42	0.09	Luteolin	0.214	0.004
(+)-Dihydrokaempferol	$C_{15}H_{12}O_{6}$	-0.07	288.06337	287.05609	6.128	0.08	Luteolin	0.199	0.003
Quercetin dihydroxybenzoyl hexoside	$C_{28}H_{24}O_{15}$	0.06	600.11155	599.10428	5.39	0.07	Luteolin	0.185	0.004
Kaempferol 3-glucoside	$C_{21}H_{20}O_{11}$	-0.84	448.10019	447.09291	5.323	0.07	Luteolin	0.182	0.002
Quercetin 3-3''-acetyl arabinofuranoside	$C_{22}H_{20}O_{12}$	0.7	476.09581	475.08853	6.84	0.07	Luteolin	0.177	0.003
Isoquercetin isomer	$C_{21}H_{20}O_{12}$	-0.53	464.09523	463.08795	5.686	0.06	Luteolin	0.159	0.004

Taxifolin 3-acetate	$C_{17}H_{14}O_8$	-0.28	346.06877	345.06149	8.221	0.05	Luteolin	0.116	0.001
Quercetin deoxyhexosyl-acetylhexoside	$C_{29}H_{32}O_{17}$	0.06	652.16399	651.15671	5.895	0.04	Luteolin	0.111	0.001
Rhamnetin hexoside	$C_{22}H_{22}O_{12}$	0.39	478.11131	477.10404	8.705	0.03	Luteolin	0.086	0.002
Kaempferol 3-arabinoside	$C_{20}H_{18}O_{10}$	-0.33	418.08986	417.08258	5.976	0.03	Luteolin	0.081	0.001
Myricetin	$C_{15}H_{10}O_8$	-0.05	318.03755	317.03027	6.123	0.03	Luteolin	0.078	0.001
Quercetin isomer	$C_{15}H_{10}O_7$	-0.63	302.04246	301.03519	6.258	0.03	Luteolin	0.072	0.002
Kaempferol coumaroyl hexoside	$C_{30}H_{26}O_{13}$	1.03	594.13795	593.13068	7.746	0.03	Luteolin	0.068	0.001
Quercetin galloyl hexoside	$C_{28}H_{24}O_{16}$	0.24	616.10658	615.0993	4.525	0.02	Luteolin	0.060	0.001
Kaempferol 3-O-neohesperidoside	$C_{27}H_{30}O_{15}$	0.09	594.15852	593.15125	4.995	0.02	Luteolin	0.054	0.001
Dihydromyricetin	$C_{15}H_{12}O_8$	-0.63	320.05302	319.04585	4.228	0.02	Luteolin	0.046	0.002
Dihydromyricetin isomer	$C_{15}H_{12}O_8$	-0.13	320.05318	319.04591	3.952	0.02	Luteolin	0.037	0.002
Myricetin coumaroyl hexoside	$C_{30}H_{26}O_{15}$	0.5	626.12748	625.12021	6.55	0.01	Luteolin	0.029	0.003
**Flavanones**									
Pinobanksin 3-propionate	$C_{18}H_{16}O_6$	-0.17	328.09463	327.08736	13.838	0.45	Naringenin	1.127	0.007
8 C Glucopyranosyleriodictyol	$C_{21}H_{22}O_{11}$	-0.57	450.11596	449.10867	4.167	0.23	Naringenin	0.571	0.021
(R)-Naringenin	$C_{15}H_{12}O_5$	0.47	272.0686	271.06133	8.955	0.07	Naringenin	0.238	0.001
Persicogenin hexoside	$C_{23}H_{26}O_{11}$	-0.1	478.14746	477.14017	5.529	0.03	Naringenin	0.073	0.001
(±)-Naringenin isomer	$C_{15}H_{12}O_5$	0.53	272.06862	271.06134	8.374	0.01	Naringenin	0.040	0.001
**Flavones**									
Palustrinoside	$C_{22}H_{20}O_{11}$	-0.63	460.10027	459.09299	5.697	0.32	Luteolin	0.796	0.008
Luteolin	$C_{15}H_{10}O_6$	0.39	286.04785	285.04057	8.935	0.10	Luteolin	0.263	0.002

Diosmetin	$C_{16}H_{12}O_6$	0.17	300.06344	299.05617	10.709	0.10	Luteolin	0.255	0.005
3-Methoxyluteolin	$C_{16}H_{12}O_7$	0.34	316.05841	315.05113	9.289	0.05	Luteolin	0.128	0.002
2"-O-*p*-Hydroxybenzoylorientin	$C_{28}H_{24}O_{13}$	0.35	568.12189	567.11472	6.749	0.05	Luteolin	0.116	0.002
Afzelin	$C_{21}H_{20}O_{10}$	-0.86	432.10528	431.09799	6.213	0.05	Luteolin	0.114	0.001
Sideritoflavone	$C_{18}H_{16}O_8$	-0.55	360.08432	359.07704	11.353	0.03	Luteolin	0.081	0.001
3-Methoxyluteolin isomer	$C_{16}H_{12}O_7$	0.2	316.05836	315.05109	10.833	0.03	Luteolin	0.076	0.003
**Isoflavones**									
3’-Methoxy puerarin	$C_{22}H_{22}O_{10}$	0.16	446.12137	445.11409	6.753	0.02	Daidzin	12.802	0.244
Genistein	$C_{15}H_{10}O_5$	0.57	270.05298	269.0457	8.691	0.02	Daidzein	0.091	0.003
**Other flavonoids**									
Dichotosinin	$C_{24}H_{30}O_{10}$	-0.8	478.18352	523.18179	4.724	0.04			
**Phenolic acids and derivatives**									
Homovanillic acid	$C_9H_{10}O_4$	-0.77	182.05777	181.05049	5.699	1.26	3,5-Dihydroxybenzoic acid	36.258	0.693
Protocatechuic aldehyde	$C_7H_6O_3$	-0.91	138.03157	137.02429	2.994	1.22	3,5-Dihydroxybenzoic acid	35.227	0.435
1-O-Vanilloyl-beta-D-glucose	$C_{14}H_{18}O_9$	-0.77	330.09483	329.08756	3.832	0.99	3,5-Dihydroxybenzoic acid	28.572	0.338
1-O-Vanilloyl-beta-D-glucose isomer	$C_{14}H_{18}O_9$	-0.99	330.09475	329.08751	3.244	0.92	3,5-Dihydroxybenzoic acid	26.547	0.206
3,5-Dihydroxybenzoic acid	$C_7H_6O_4$	-0.89	154.02647	153.0192	1.928	0.90	3,5-Dihydroxybenzoic acid	25.969	0.416
Dihydroxybenzoic acid hexoside	$C_{13}H_{16}O_9$	-0.81	316.07918	315.0719	1.545	0.65	3,5-Dihydroxybenzoic acid	18.940	0.123
Vanillyl hexoside	$C_{14}H_{20}O_8$	-0.75	316.11558	315.10832	1.779	0.58	3,5-Dihydroxybenzoic acid	16.821	0.204
Salicylic Acid hexoside	$C_{13}H_{16}O_8$	-0.64	300.08432	299.07706	1.832	0.53	3,5-Dihydroxybenzoic acid	15.159	0.262
Gallic acid	$C_7H_6O_5$	-0.86	170.02138	169.0141	1.691	0.45	3,5-Dihydroxybenzoic acid	12.910	0.352

Galloyl hexoside	$C_{13}H_{16}O_{10}$	-0.62	332.07414	331.06686	1.04	0.41	3,5-Dihydroxybenzoic acid	11.944	0.164
Pyrogallol hexuronide	$C_{12}H_{14}O_9$	-0.31	302.06369	301.05641	1.281	0.18	3,5-Dihydroxybenzoic acid	5.336	0.057
Salicylic Acid hexoside isomer	$C_{13}H_{16}O_8$	-0.8	300.08428	299.07703	1.23	0.17	3,5-Dihydroxybenzoic acid	4.843	0.007
Gallic acid isomer	$C_7H_6O_5$	-0.69	170.02141	169.01413	1.225	0.17	3,5-Dihydroxybenzoic acid	4.810	0.089
3-Hydroxybenzoic acid	$C_7H_6O_3$	-0.69	138.0316	137.02432	1.23	0.16	3,5-Dihydroxybenzoic acid	4.624	0.056
Protocatechuic acid	$C_7H_6O_4$	-0.91	154.02647	153.01923	1.714	0.12	3,5-Dihydroxybenzoic acid	3.441	0.092
Dihydroxybenzoic hydroxybenzoic acid hexoside	$C_{20}H_{20}O_{11}$	-0.16	436.10049	435.09322	7.366	0.11	3,5-Dihydroxybenzoic acid	3.147	0.029
Benzoic acid	$C_7H_6O_2$	-0.51	122.03672	121.02944	3.776	0.09	3,5-Dihydroxybenzoic acid	2.610	0.030
Vanillyl alcohol	$C_8H_{10}O_3$	-0.59	154.0629	153.05563	1.778	0.09	3,5-Dihydroxybenzoic acid	2.590	0.001
Ethyl vanillate hexoside	$C_{16}H_{22}O_9$	-1.47	358.12585	357.11858	3.232	0.07	3,5-Dihydroxybenzoic acid	1.869	0.073
Vanillyl hexoside isomer	$C_{14}H_{20}O_8$	-0.81	316.11556	315.10834	1.638	0.05	3,5-Dihydroxybenzoic acid	1.546	0.001
Pyrogallol	$C_6H_6O_3$	-0.54	126.03163	125.02435	1.221	0.02	3,5-Dihydroxybenzoic acid	0.690	0.012
Gastrodigenin	$C_7H_8O_2$	-0.63	124.05235	123.04507	1.761	0.02	3,5-Dihydroxybenzoic acid	0.654	0.026
Gastrodigenin isomer	$C_7H_8O_2$	-0.57	124.05236	123.04508	2.187	0.02	3,5-Dihydroxybenzoic acid	0.605	0.028
Dihydroxybenzoic acid hexoside isomer	$C_{13}H_{16}O_9$	0.5	316.07959	315.07231	1.798	0.02	3,5-Dihydroxybenzoic acid	0.497	0.015
Gastrodigenin isomer	$C_7H_8O_2$	-0.02	124.05243	123.04515	1.889	0.02	3,5-Dihydroxybenzoic acid	0.446	0.020
Ethyl gallate	$C_9H_{10}O_5$	-0.38	198.05275	197.04547	1.307	0.01	3,5-Dihydroxybenzoic acid	0.315	0.028
**Hydroxycinnamic acids and derivatives**									
1-Caffeoylquinic acid	$C_{15}H_{16}O_7$	-1.99	308.08899	353.08719	2.449	2.24	Chlorogenic acid	171.559	1.760
Chlorogenic acid	$C_{16}H_{18}O_9$	-1.81	354.09444	353.08714	3.225	1.51	Chlorogenic acid	116.202	0.198
1-O-Feruloyl-beta-D-glucose	$C_{16}H_{20}O_9$	-1.52	356.11019	355.10292	3.649	0.28	Caffeic acid	3.911	0.027

1-Caffeoyl-beta-D-glucose	$C_{15}H_{18}O_9$	-1.29	342.09464	341.08736	2.74	0.28	Caffeic acid	3.748	0.229
Coumaroylquinic acid	$C_{16}H_{18}O_8$	-0.39	338.10004	337.09276	3.694	0.26	Caffeic acid	3.587	0.085
Caffeic acid	$C_9H_8O_4$	-0.86	180.0421	179.03483	3.421	0.17	Caffeic acid	2.356	0.059
Coumaroyl hexoside	$C_{15}H_{18}O_8$	-0.67	326.09995	325.0927	3.21	0.14	Caffeic acid	2.003	0.004
(E)-*p*-Coumaric acid	$C_9H_8O_3$	-0.4	164.04728	163.04	4.241	0.12	Caffeic acid	1.693	0.058
Coumaroylquinic acid isomer	$C_{16}H_{18}O_8$	-0.41	338.10003	337.09276	3.065	0.11	Caffeic acid	1.592	0.010
Abietin	$C_{16}H_{22}O_8$	-1.31	342.13102	341.12374	3.647	0.11	Caffeic acid	1.532	0.053
Coumaroyl hexoside isomer	$C_{15}H_{18}O_8$	-0.58	326.09998	325.0927	3.517	0.10	Caffeic acid	1.308	0.034
Sinapinate	$C_{11}H_{12}O_5$	-0.15	224.06844	223.06117	2.773	0.08	Caffeic acid	1.070	0.077
Dihydroxyferuloyl pentoside	$C_{15}H_{18}O_{10}$	-0.43	358.08984	357.08256	4.633	0.08	Caffeic acid	1.052	0.061
(E)-*p*-Coumaric acid isomer	$C_9H_8O_3$	-0.68	164.04723	163.03996	3.209	0.08	Caffeic acid	1.063	0.033
3-O-Feruloyl-D-quinic acid	$C_{17}H_{20}O_9$	-1.2	368.11029	367.10301	3.423	0.05	Caffeic acid	0.727	0.015
Coumaroyl hexoside isomer	$C_{15}H_{18}O_8$	-0.62	326.09997	371.09809	2.579	0.05	Caffeic acid	0.632	0.071
trans-5-O-(4-Coumaroyl)-D-quinic acid	$C_{16}H_{18}O_8$	-0.3	338.10007	337.09279	4.116	0.05	Caffeic acid	0.642	0.010
p-Coumaric acid ethyl ester	$C_{11}H_{12}O_3$	0.03	192.07865	191.07137	9.037	0.04	Caffeic acid	0.582	0.011
6-O-Sinapoyl-D-glucono-1,5-lactone	$C_{17}H_{20}O_{10}$	-0.35	384.10551	429.10376	4.2	0.01	Caffeic acid	0.202	0.003
Coumaroyl quinic derivative	$C_{26}H_{28}O_{12}$	0.84	532.15852	531.15125	6.717	0.01	Caffeic acid	0.189	0.006
Caffeoylglycolic acid methyl ester	$C_{12}H_{12}O_6$	-0.57	252.06325	251.05597	3.4	0.01	Caffeic acid	0.149	0.006
trans-*p*-Coumaraldehyde	$C_9H_8O_2$	-0.48	148.05236	147.04508	2.862	0.01	Caffeic acid	0.097	0.001
**Coumarins**									
Fraxin	$C_{16}H_{18}O_{10}$	-2.01	370.08925	369.08197	3.742	9.07			

Aesculin	$C_{15}H_{16}O_9$	-1.82	340.07881	339.07152	3.136	4.19			
Fraxetin	$C_{10}H_8O_5$	-1.12	208.03694	207.02966	4.264	3.08			
5,7-Dihydroxychromone	$C_9H_6O_4$	-1.19	178.0264	177.01912	3.59	2.99			
Feruloyl aesculin	$C_{25}H_{24}O_{12}$	-0.39	516.12657	515.11929	6.878	0.73			
Scopolin	$C_{16}H_{18}O_9$	-1.59	354.09452	399.0927	3.647	0.71			
Aesuletin derivative	$C_{21}H_{24}O_{13}$	-0.79	484.12131	483.11403	4.124	0.60			
Coumaroyl aesculin	$C_{24}H_{22}O_{11}$	-0.44	486.116	485.10872	6.599	0.60			
3-Acetyl-7-methoxychromen-2-one	$C_{12}H_{10}O_4$	-0.71	218.05775	217.05048	4.319	0.60			
Scopoletin	$C_{10}H_8O_4$	-0.88	192.04209	191.03481	3.647	0.40			
Caffeoyl aesculin	$C_{24}H_{22}O_{12}$	-0.39	502.11093	501.10365	5.621	0.32			
Aesculin derivative	$C_{31}H_{34}O_{18}$	-0.66	694.17405	693.16678	3.16	0.31			
Peniisocoumarin I	$C_{13}H_{14}O_7$	-1.49	282.07353	341.08737	3.043	0.19			
6,7-Dihydroxycoumarin	$C_9H_6O_4$	-0.69	178.02648	177.01921	3.149	0.13			
Aesculin isomer	$C_{15}H_{16}O_9$	-1.24	340.07901	339.07173	3.861	0.11			
Fraxin isomer	$C_{16}H_{18}O_{10}$	-1.42	370.08947	369.0822	3.293	0.06			
Fraxetin isomer	$C_{10}H_8O_5$	-0.81	208.03701	207.02973	3.874	0.05			
Scopolin isomer	$C_{16}H_{18}O_9$	-0.76	354.09481	399.09306	4.144	0.02			
Isofraxidin	$C_{11}H_{10}O_5$	0.07	222.05284	221.04556	5.445	0.02			
**Stilbenes**									
Piceid	$C_{20}H_{22}O_8$	-1.28	390.13097	435.12915	4.91	2.65	3,3',4',5-Tetrahydroxystilbene	18.207	0.255
Resveratrol	$C_{14}H_{12}O_3$	-0.51	228.07853	227.07125	4.91	0.37	3,3',4',5-Tetrahydroxystilbene	2.53	0.014

Piceid isomer	$C_{20}H_{22}O_8$	-0.63	390.13122	389.1239	5.778	0.17	3,3',4',5-Tetrahydroxystilbene	1.178	0.021
Astringin	$C_{20}H_{22}O_9$	-0.1	406.12634	405.11905	6.359	0.02	3,3',4',5-Tetrahydroxystilbene	0.142	0.003
trans-Resveratrol	$C_{14}H_{12}O_3$	0.19	228.07869	227.07141	7.073	0.02	3,3',4',5-Tetrahydroxystilbene	0.122	0.001
trans-Resveratrol isomer	$C_{14}H_{12}O_3$	0.59	228.07878	227.0715	7.806	0.01	3,3',4',5-Tetrahydroxystilbene	0.080	0.001
**Vitamins**									
(+)-Riboflavin	$C_{17}H_{20}N_4O_6$	-4.73	376.13651	375.12926	1.047	0.02			
Vitamin C	$C_6H_8O_6$	-0.55	176.03199	175.02471	3.979	0.00			
**Fatty acids and derivatives**									
10,16-Dihydroxyhexadecanoic acid	$C_{16}H_{32}O_4$	-0.33	288.22996	287.22269	9.143	0.77			
(10E)-9,13-Dihydroxy-10-octadecenoic acid	$C_{18}H_{34}O_4$	-0.68	314.2455	313.23822	12.379	0.76			
Threonic acid, L-	$C_4H_8O_5$	-1.05	136.03703	135.02975	0.709	0.69			
(10E,12E)-9-Hydroxy-10,12-octadecadienoic acid	$C_{18}H_{32}O_3$	-0.44	296.23501	295.22774	14.002	0.50			
9-HOTE	$C_{18}H_{30}O_3$	-0.52	294.21934	293.21207	13.54	0.47			
(9E)-8,11,12-Trihydroxy-9-octadecenoic acid	$C_{18}H_{34}O_5$	0.15	330.24067	329.2334	8.858	0.40			
(9S,10E,12S,13S,15Z)-9,12,13-Trihydroxy-10,15-octadecadienoic acid	$C_{18}H_{32}O_5$	0	328.22497	327.2177	8.175	0.31			
(9S,10R)-9,10,18-Trihydroxyoctadecanoic acid	$C_{18}H_{36}O_5$	0.26	332.25636	331.24908	8.51	0.16			
9-KODE	$C_{18}H_{30}O_3$	0.11	294.21953	293.21225	14.624	0.09			
10-oxoundecanoic acid	$C_{11}H_{20}O_3$	-0.27	200.14119	259.15504	7.444	0.09			
(3R,5R)-3-O-β-D-mannosyl-3,5-dihydrodecanoic acid	$C_{16}H_{30}O_9$	-1.35	366.18849	365.18121	3.729	0.07			
Suberic acid	$C_8H_{14}O_4$	-0.48	174.08912	173.08185	4.072	0.06			

Methyl 9-(alpha-D-galactosyloxy)nonanoate	$C_{16} H_{30} O_8$	-1.16	350.19366	395.19186	3.967	0.03
3-Hydroxyheptanoic acid	$C_7 H_{14} O_3$	-0.5	146.09422	145.08694	4.937	0.02
Rel-2alpha,3alpha,23-trihydroxy-19-oxo-18,19-seco-urs-11,13(18)-dien-28-oic acid	$C_{30}H_{46}O_6$	0.34	502.32961	501.32233	11.837	0.02
1-Heptadecanoyl-rac-glycerol	$C_{20}H_{40}O_4$	-0.44	344.29251	343.28523	13.968	0.02
7-Methyl-3-oxo-6-octenoic acid	$C_9H_{14}O_3$	0.43	170.09437	169.08709	8.572	0.02
Glycol stearate	$C_{20}H_{40}O_3$	0.32	328.29785	327.29057	15.962	0.02
6-Oxohexanoic acid	$C_6H_{10}O_3$	-0.41	130.06294	129.05566	1.324	0.02
(8E)-8-Heptadecenedioic acid	$C_{17}H_{30}O_4$	0.12	298.21445	297.20717	10.848	0.02
Linoleic acid	$C_{18}H_{32}O_2$	0.12	280.24026	279.23299	16.084	0.01
8-Hydroxyhexadecanedioic acid	$C_{16}H_{30}O_5$	0.49	302.20947	301.20219	8.973	0.01
11-Dodecynoic acid	$C_{12}H_{20}O_2$	0.43	196.14641	195.13914	8.563	0.01
9-Methoxy-9-oxononanoic acid	$C_{10}H_{18}O_4$	-0.25	202.12046	201.1132	4.411	0.01
Thapsic acid	$C_{16}H_{30}O_4$	0.45	286.21454	285.20726	10.445	0.01
Methyl 3-hydroxyhexadecanoate	$C_{17}H_{34}O_3$	0.31	286.25088	285.24361	14.268	0.01
Linolenelaidic acid	$C_{18}H_{30}O_2$	-0.67	278.22439	277.21712	15.698	0.01
9-Oxononanoic acid	$C_9H_{16}O_3$	-0.04	172.10994	171.10266	10.057	0.00
**Sugars and derivatives**						
Benzyl Î²-primeveroside	$C_{18}H_{26}O_{10}$	-1.45	402.15201	447.15021	3.344	1.05
Salidroside	$C_{14}H_{20}O_7$	-1.94	300.12032	345.11847	1.695	1.03
Phenethyl Î²-primeveroside	$C_{19}H_{28}O_{10}$	-1.09	416.16779	461.16597	3.976	1.02

D-(-)-Arabinose	$C_5H_{10}O_5$	-1.39	150.05261	195.05081	0.7	0.90
D-(+)-Glucose	$C_6H_{12}O_6$	-1.15	180.06318	179.05592	0.701	0.84
Methyl 2-O-beta-L-arabinofuranosyl-beta-L-arabinofuranoside	$C_{11}H_{20}O_9$	-0.83	296.11049	295.10321	0.998	0.43
2-Hydroxy-2-(4-methyl-2-oxocyclohexyl)propyl hexopyranoside	$C_{16}H_{28}O_8$	-1.33	348.17796	393.17615	3.674	0.41
2-Hydroxy-2-(4-methyl-2-oxocyclohexyl)propyl hexopyranoside isomer	$C_{16}H_{28}O_8$	-1.31	348.17796	393.17615	3.792	0.37
D-3-Deoxyglucosone	$C_6H_{10}O_5$	-0.81	162.05269	161.04541	1.705	0.36
3,7-Dimethyl-1,6-octadien-3-yl 6-O-beta-D-xylopyranosyl-beta-D-glucopyranoside	$C_{21}H_{36}O_{10}$	-0.44	448.23065	493.22884	5.899	0.35
Arbutin	$C_{12}H_{16}O_7$	-0.76	272.08939	317.08759	1.019	0.28
(-)-Rhododendrin	$C_{16}H_{24}O_7$	-1.01	328.15187	327.14467	3.715	0.17
Hinokitiol hexoside	$C_{16}H_{22}O_7$	-1.42	326.13609	371.13428	3.776	0.16
4-Allyl-2-methoxyphenyl 6-O-beta-D-xylopyranosyl-beta-D-glucopyranoside	$C_{21}H_{30}O_{11}$	-0.33	458.17866	503.17686	5.713	0.16
2-Phenylethyl 6-O-(6-deoxy-alpha-L-mannopyranosyl)-beta-D-glucopyranoside	$C_{20}H_{30}O_{10}$	-0.69	430.1836	475.18179	4.242	0.14
1,5-Anhydro-D-fructose	$C_6H_{10}O_5$	-0.73	162.0527	161.04543	0.996	0.14
alpha-L-Rhap-(1->3)-D-ribitol	$C_{11}H_{22}O_9$	-1.15	298.12604	297.11885	0.738	0.13
2,6-Dideoxy-D-arabino-hexopyranose	$C_6H_{12}O_4$	-0.89	148.07343	207.08728	0.944	0.10
Ethyl 3-(beta-D-glucopyranosyloxy)-butanoate	$C_{12}H_{22}O_8$	-0.36	294.13136	293.12408	2.706	0.10
4-Allyl-2-methoxyphenyl 6-O-beta-D-xylopyranosyl-beta-D-glucopyranoside	$C_{21}H_{30}O_{11}$	-0.97	458.17837	503.17657	3.757	0.08
Benzyl Î²-primeveroside	$C_{18}H_{26}O_{10}$	-0.95	402.15221	447.15042	3.422	0.08
D-(+)-Glucose	$C_6H_{12}O_6$	-0.62	180.06328	179.056	4.305	0.07

δ-Gluconic acid δ-lactone	$C_6H_{10}O_6$	-1.11	178.04754	177.04027	0.74	0.07			
6-Deoxy-alpha-L-galactopyranosyl-(1->2)-[alpha-D-galactopyranosyl-(1->3)]-D-galactitol	$C_{18}H_{34}O_{15}$	-1.05	490.18925	489.18198	0.732	0.06			
Zizybeoside I	$C_{19}H_{28}O_{11}$	-0.44	432.16297	477.16112	3.101	0.05			
Saccharumoside C	$C_{19}H_{26}O_{13}$	-0.53	462.1371	443.11933	3.647	0.04			
Isosalicin	$C_{13}H_{18}O_7$	-0.28	286.10517	285.0979	2.822	0.03			
Butyl 3-(beta-D-glucopyranosyloxy)butanoate	$C_{14}H_{26}O_8$	-0.49	322.16261	321.15533	3.486	0.02			
D-(−)-Salicin	$C_{13}H_{18}O_7$	-0.48	286.10512	331.10329	1.121	0.02			
**Organic acids**									
Arabic acid	$C_5H_{10}O_6$	-1.07	166.04756	165.04028	0.699	1.01			
Citric acid	$C_6H_8O_7$	-0.98	192.02681	191.01954	0.855	0.79			
(E)-Aconitic acid	$C_6H_6O_6$	-0.7	174.01632	173.00904	2.183	0.06			
3-Methylazelaic acid	$C_{10}H_{18}O_4$	-0.25	202.12046	201.11318	5.789	0.02			
DL-Malic acid	$C_4H_6O_5$	-0.82	134.02141	133.01414	0.919	0.01			
3-Methylglutarate	$C_6H_{10}O_4$	-0.87	146.05778	337.11379	0.916	0.01			
**Terpenes and derivatives**									
20S,24S-Dihydroxydammer-25-en-3-one	$C_{30}H_{50}O_3$	-1.08	458.3755	503.37369	16.552	1.54	Asiatic acid	52.554	0.522
Nepetaside	$C_{16}H_{26}O_8$	-1.58	346.16222	345.15492	3.49	1.22	Agnuside	5.628	0.071
Ursolic acid	$C_{30}H_{48}O_3$	-0.81	456.35998	455.35269	15.681	0.90	Asiatic acid	30.746	0.496
Deoxyloganic acid	$C_{16}H_{24}O_9$	-1.48	360.1415	359.13422	2.948	0.36	Agnuside	1.659	0.030
Asiatic acid	$C_{30}H_{48}O_5$	-0.88	488.34974	487.34245	12.197	0.34	Asiatic acid	11.547	0.011

Corosolic acid	$C_{30}H_{48}O_4$	-0.79	472.35489	471.34761	16.951	0.32	Asiatic acid	11.072	0.060
Corosolic acid isomer	$C_{30}H_{48}O_4$	-0.56	472.355	471.34772	17.132	0.28	Asiatic acid	9.623	0.186
Ginsenoside Rh2	$C_{36}H_{62}O_8$	-0.14	622.44438	621.4371	16.443	0.25	Asiatic acid	8.422	0.002
beta-Elemonic acid	$C_{30}H_{46}O_3$	-0.33	454.34455	453.33725	15.491	0.19	Asiatic acid	6.399	0.010
Methyl (1S,4aR,5R,7aR)-1-(beta-D-glucopyranosyloxy)-7-(hydroxymethyl)-5-[(2S)-2-hydroxypropoxy]-1,4a,5,7a-tetrahydrocyclopenta[c]pyran-4-carboxylate	$C_{20}H_{30}O_{12}$	-1.2	462.17317	461.16589	1.702	0.17	Agnuside	0.778	0.016
Ursolic acid isomer	$C_{30}H_{48}O_3$	-0.82	456.35997	501.35817	17.065	0.13	Asiatic acid	4.578	0.055
Methyl (1S,4aR,5R,7aR)-5-ethoxy-1-(beta-D-glucopyranosyloxy)-7-(hydroxymethyl)-1,4a,5,7a-tetrahydrocyclopenta[c]pyran-4-carboxylate	$C_{19} H_{28} O_{11}$	-0.96	432.16275	477.16096	1.745	0.10	Agnuside	0.479	0.008
Agnuside	$C_{22}H_{26}O_{11}$	-0.21	466.14741	465.14011	4.38	0.09	Agnuside	0.430	0.001
7-Deoxyloganin	$C_{17}H_{26}O_9$	-1.19	374.15724	373.14995	3.573	0.08	Agnuside	0.351	0.004
Tomentosic acid	$C_{30}H_{48}O_6$	0.36	504.34527	503.33795	9.955	0.05	Asiatic acid	1.777	0.004
Platanic acid	$C_{29}H_{46}O_4$	-0.5	458.33938	457.33211	13.627	0.05	Asiatic acid	1.680	0.019
Demethyloleuropein	$C_{24}H_{30}O_{13}$	-0.64	526.1683	525.16104	3.361	0.05	Asiatic acid	0.215	0.001
Loganin	$C_{17}H_{26}O_{10}$	-1.23	390.15212	371.13435	2.643	0.04	Agnuside	0.200	0.004
Soyasapogenol A	$C_{30}H_{50}O_4$	-0.04	474.37089	519.36908	15.239	0.04	Asiatic acid	1.203	0.004
Tsugaric acid A	$C_{32}H_{50}O_4$	-0.01	498.37091	497.36363	17.011	0.01	Asiatic acid	0.500	0.006
(±)-(2E)-Abscisic acid	$C_{15}H_{20}O_4$	0.32	264.13624	263.12897	6.645	0.00	Abscisic acid	0.109	7.0999E-05
**Ketones**									
Diacetylphloroglucinol	$C_{10}H_{10}O_5$	-1.56	210.0525	191.03483	3.854	0.05			

Ellagic acid	$C_{14}H_6O_8$	0.01	302.00627	300.99899	4.535	0.01
Triacetic acid	$C_6H_8O_4$	-0.53	144.04218	143.0349	1.54	0.01
**Xanthones**						
Swertianolin	$C_{20}H_{20}O_{11}$	0.12	436.10061	435.09334	4.605	0.01
**Chalcones**						
Neobavachalcone	$C_{17}H_{14}O_5$	-0.78	298.08389	297.07661	13.299	1.29
Phloretin	$C_{15}H_{14}O_5$	0.15	274.08417	273.07689	8.865	0.02
**Other compounds**						
Pisatin	$C_{17}H_{14}O_6$	-0.44	314.0789	313.07162	12.746	0.48
Gingerglycolipid A	$C_{33}H_{56}O_{14}$	-0.57	676.36662	721.36481	12.905	0.29
Resorcinol monoacetate	$C_8H_8O_3$	-0.64	152.04725	151.03997	3.388	0.11
Swerilactone L, (rel)-	$C_{12}H_{14}O_5$	-0.95	238.0839	237.07662	3.301	0.05
2-Phenylethyl Î²-D-glucopyranosiduronic acid	$C_{14}H_{18}O_7$	-1.33	298.10485	343.10303	3.038	0.04
2,4-Toluenediol	$C_7H_8O_2$	-0.05	124.05242	123.04515	4.994	0.02

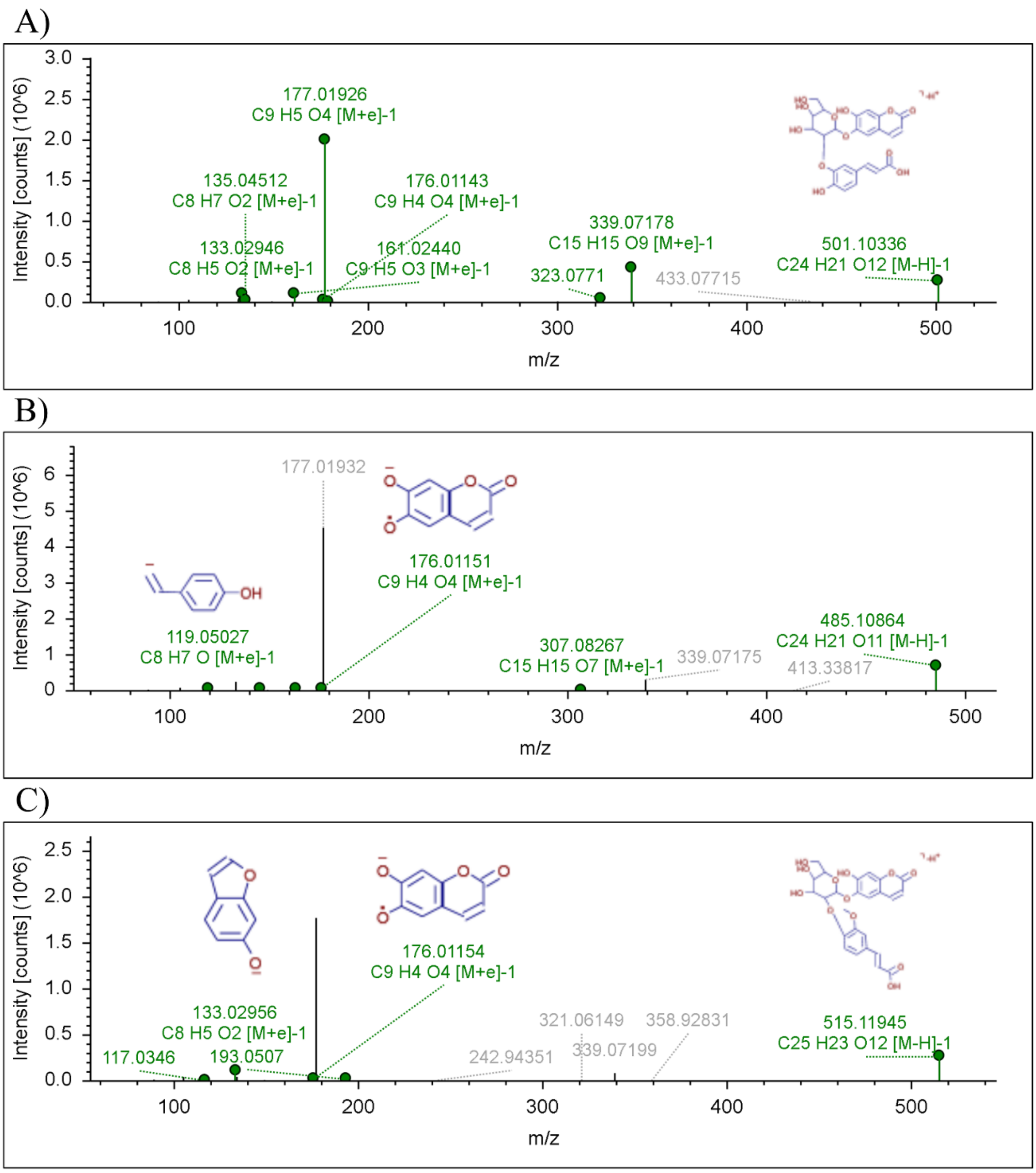

FIGURE S1 Tandem mass spectra of caffeoyl aesculin (A), coumaroyl aesculin (B), and feruloyl aesculin (C). The chemical structure of each compound was imported in Compound Discoverer upon a dedicated procedure developed in ChemDraw software. Each fragmentation pattern was screened through the FISh scoring node according to the most intense product ions and the respective chemical structure.
